# Muscle Function, Muscle Disease, and Positron Emission Tomography-Computed Tomography: A Narrative Review

**DOI:** 10.7759/cureus.79857

**Published:** 2025-02-28

**Authors:** Shinji Yamamoto, Yukinori Okada

**Affiliations:** 1 Department of Radiological Technology, Japan Community Healthcare Organization (JCHO) Tokyo Yamate Medical Center, Tokyo, JPN; 2 Department of Radiology, Tokyo Medical University Hospital, Tokyo, JPN

**Keywords:** 18f-fdg, dermatomyositis, dystonia, muscle, pet, polymyositis

## Abstract

^18^F-fluorodeoxyglucose (^18^F-FDG) is a radiopharmaceutical that exhibits glucose-like kinetics and is used in positron emission tomography (PET). ^18^F-FDG is used for cancer diagnosis in clinical practice. However, ^18^F-FDG uptake is also observed in normal organs, such as the brain, liver, and heart, with high glucose consumption. Moreover, ^18^F-FDG uptake is also observed in muscles, where its accumulation and radioactivity reflect muscle activity. Dystonia is characterized by excessive muscle movement. Recently, ^18^F-FDG and technetium-99m-methoxyisobutyl isocyanide ([99mTc]MIBI) have been used for the diagnosis and botulinum toxin therapy evaluation of dystonia.

This review aimed to summarize the utility of ^18^F-FDG-PET for the evaluation of muscle activity and diagnosis of muscle diseases such as dystonia, polymyositis, dermatomyositis, and polymyalgia rheumatica.

## Introduction and background

The muscle is a large body organ. In clinical practice, the evaluation of muscle function is important. Muscle function, in particular, is related not only to disease but also to sports. Ultrasonography (US), particularly elastography, and magnetic resonance imaging (MRI) are used to evaluate muscle function. During US, shear waves propagate faster in hard materials and slower in soft materials. From the speed of the shear wave, we can evaluate the stiffness of organs such as muscles. Elastography, a form of US, is used for muscle evaluation, particularly for high-speed dynamic performance [1]. However, US has certain limitations, including interpreter subjectivity. Moreover, whole-body muscle examination with US is difficult, as the number of muscles that can be evaluated simultaneously is limited. Similarly, MRI can be used to evaluate the shape and function of muscles [2] and has good contrast resolution. T2-weighted imaging adequately reflects inflammation and edema, and the utility of T2 mapping for dermatomyositis/polymyositis [3] and muscular dystrophy [4] has been reported. Moreover, the utility of intravoxel incoherent motion (IVIM), a part of diffusion-weighted imaging (DWI), has also been reported [4]. Diffusion-weighted whole-body imaging with background suppression (DWIBS) is used to evaluate whole body organs [5]. However, quantitative evaluation using DWIBS is limited. In DWIBS, the apparent diffusion coefficient (ADC) is used as a quantitative index; however, the utility of the ADC value has not been established [6].

^18^F-fluorodeoxyglucose (^18^F-FDG) with positron emission tomography (PET), which has the disadvantage of radiation exposure, is often used to evaluate muscle function. ^18^F-FDG exhibits glucose-like properties and enters the muscle through the glucose transporter 4. For these reasons, ^18^F-FDG PET is used to evaluate muscle function, activity, and disease through standard uptake value (SUV) quantitative evaluation of the radioactivity of organs due to accumulated ^18^F-FDG. Therefore, ^18^F-FDG PET and scintigraphy are useful tools for the evaluation of muscle function.

## Review

We used PubMed, corrected the original article, and wrote a narrative review on muscle function, shoulder function, dystonia, polymyositis, dermatomyositis, and polymyalgia rheumatica. In this narrative review, we used the following keywords: PET, scintigraphy, muscle function, shoulder function, dystonia, polymyositis/dermatomyositis, and polymyalgia rheumatica. Original articles written in English between 1999 and 2025 were selected. The following manuscript types were excluded: short communications, articles not written in English (such as in Japanese), case reports, review articles, meta-analysis articles, manuscripts with content that was difficult to understand, and manuscripts evaluating two or more similar diseases (for example, in the polymyalgia rheumatica session, we did not use articles with polymyalgia rheumatica and other related diseases such as vasculitis collagen disease), unless these articles were deemed crucial. Additionally, we used a case report related to this research written by author Okada to explain the utility of FDG-PET [7].

Muscle function and FDG

Initial Research

In Japan, there are some reports on the accumulation of FDG in muscles. One report investigated the relationship between muscle exercise and FDG-PET imaging. It showed that FDG uptake increased in the gastrocnemius and soleus muscles after walking in a 22-year-old healthy male participant [8].

Exercise

In a report that evaluated seven healthy, exercise-experienced male adults aged between 19 and 23 years, the sole muscles showed the highest metabolic activation during running [9]. In 17 healthy adults, FDG uptake in the arm muscles correlated with the exercise load at the biceps brachii and brachialis [10]. In a study including seven male participants, exercise increased glucose uptake by 30% to 55% [11]. In a study including 20 healthy male participants, accumulation in the iliac muscles and the anterior part of the thighs increased during exercise load [12]. In 17 healthy male participants (11 walking, six resting), muscle FDG uptake in the lower extremities during walking increased significantly [13]. In six healthy male participants and six control male participants, after sprinting for 10 minutes at full speed, FDG uptake increased in the anterior and posterior legs in the control group [14]. The accumulation of FDG in the muscles differs when running versus sprinting. In a previous study, seven out of 13 healthy male participants were subjected to an exercise load with FDG administered after running for 15 min [15]. Compared to resting, running increased glucose metabolism in the tibialis anterior, peroneal, posterior tibial, and axillary muscles, extensor digitorum longus, soleus, gastrocnemius, leg extensors, leg flexors, plantar quadratus, flexor digitorum brevis, and abductor digit minim muscles. However, the gastrocnemius muscle showed a significant increase in uptake in the medial head compared to the lateral head and a small increase in uptake in the thigh and hip joints [16]. The FDG accumulation in each muscle was different. In a study including eight healthy participants who were examined (17 muscles divided into 10 equal parts), an uneven accumulation of FDG was shown in 12 out of 17 of the muscles [16]. Based on these reports, we concluded that FDG-PET is suitable for evaluating muscle function. Okada et al. compared glucose metabolism in four healthy people after performing a 30-minute walking task using flat-shaped general and cuboid bone support insoles. Using a cuboid support insole improves exercise performance, such as walking speed and strength, and demonstrates that FDG strongly accumulates in the triceps suture muscle and improves left-right symmetry [7]. In other words, the use of a cuboid bone support insole allows the function of the lower leg muscles to be exhibited satisfactorily and reduces the load [7].

Muscle contours were defined in 10 healthy male participants. The results revealed an increased FDG uptake in the soleus, gluteus maximus, vastus lateralis, medial gastrocnemius, and adductor magnus muscles. However, FDG uptake values vary widely [17]. Based on these reports, we concluded that FDG-PET is useful for evaluating muscle function. In a recent report on exercise and FDG uptake in the muscle [18], 23 female participants with obesity (body mass index >25 kg/m²) received a three-month physical exercise program, and FDG uptake was compared. In the initial PET scan, FDG uptake in the psoas muscle showed a correlation with highly sensitive C-reactive protein (CRP; r=0.61) and spleen SUVmax (r=0.435). Highly sensitive CRP is an inflammatory marker, and ^18^F-FDG uptake in the psoas muscle indicates whole-body inflammation [18].

Positron Emission Tomography-Magnetic Resonance Imaging

Recently, PET-MRI has been used in clinical practice. Ten healthy male participants were asked to perform a knee extension exercise 10 times in 60 seconds, followed by a pause, followed by a hand grip for 30 seconds, and then a pause of 30 seconds. This task was repeated eight times, and ^18^F-FDG uptake was found to correlate with MRI T2 prolongation (r = 0.71) [19]. In 21 patients who underwent transplantation after anterior cruciate ligament rupture, grafts had significantly lower metabolic activity more than two years after reconstruction than grafts that were recently transplanted [20].

Shoulders and FDG

Increased muscle mass is not limited to the lower extremities. In six healthy young male participants, the deltoid, supraspinatus, and upper subscapularis muscles showed increased uptake with arm elevation in the plane of the deltoid, supraspinatus, and superior portions of the subscapularis muscles [21]. In addition, several studies on shoulders have described the relationship between FDG uptake and exercise. In 10 healthy young male participants, changes in FDG accumulation in the middle deltoid, subscapularis, and suprascapular muscles, which are related to the shoulder joint, were observed. Full-can testing refers to supraspinatus function testing with low activity of the middle deltoid and subscapularis muscles [22]. A study of 50 adhesive capsulitis (AC) showed that increased local uptake in the rotator interval (RI) and/or interior capsule (IC) increased the likelihood of disease [23]. Furthermore, a study including 35 patients with AC identified increased accumulation in the rotator cuff space RI and anterior IC, including the axillary recess (AR), and reported that these sites were the main pathological sites of idiopathic AC [24]. In 11 patients with frozen shoulders and nine patients with impingement, a visual and semi-quantitative evaluation of shoulder FDG uptake was able to distinguish between the two conditions, with a sensitivity of 100 and a specificity of 93 [25]. This finding suggests that FDG may be useful for the diagnosis of shoulder diseases. In addition, 10 individuals showed increased accumulation in their fingertips and toes, on both the throwing and non-throwing sides, when FDG accumulation in muscles during pitching was evaluated in the article [26].

Dystonia and FDG/methoxyisobutyl isocyanide (MIBI)

Dystonia is characterized by involuntary muscle movements. Physical examinations and electromyography (EMG) are used to diagnose dystonia in routine practice; however, recent studies have reported the utility of nuclear medicine in the diagnosis of dystonia. PubMed was searched for articles related to dystonia and nuclear medicine. Below is a review of dystonia and nuclear medicine.

Fluorodeoxyglucose-Positron Emission Tomography

Fluorodeoxyglucose-PET imaging has been used to evaluate abnormal muscles through visual assessment. For instance, examination of six patients (all male, age 37±16 years) with idiopathic cervical dystonia revealed increased uptake in the dystonic muscles of all six individuals [27]. Similarly, examination of 10 patients with cervical dystonia (five male and five female patients, age 44±13 years) in another study revealed that 21 of 80 muscles were dystonic. The sensitivity, specificity, and accuracy of ^18^F-FDG PET for detection were 76% (16/21), 92% (54/59), and 88% (70/80), respectively. The corresponding EMG values were 95% (20/21), 66% (39/59), and 74% (59/80), respectively. Therefore, PET may be a more accurate imaging modality [28]. Botulinum toxin can improve the symptoms of dystonia; consequently, it has been used to treat dystonia. Fluorodeoxyglucose-PET has been used in conjunction with botulinum toxin therapy. For instance, improvement in dystonia symptoms was reported in four patients who received PET-CT-guided botulinum toxin [27]. In another study of patients with cervical dystonia, 16/32 (eight male and eight female patients, age 38.1±12.2 years) were treated with botulinum toxin through the clinical target method, and the other 16/32 patients (six male and 10 female patients, age 45.1±12.3 years) were treated with botulinum toxin through the FDG-PET-guided method. The re-injection rate six months after botulinum injection was lower in the ^18^F-FDG PET-guided method group (10 vs. three patients in the clinical target method group and the ^18^F-FDG PET-guided method group, respectively) [29]. Notably, 33 out of 47 patients with cervical dystonia (21 male and 26 female patients, age 38.23±12.05 years) were positive for FDG-PET uptake in a previous study [30]. When using a cutoff value of the total Toronto Western Spasmodic Torticollis Rating Scale (TWSTRS) of 27.5, the sensitivity and specificity were 90.9% and 64.3%, respectively, based on FDG-PET positive results [30]. The administration of botulinum toxin in 78 cervical dystonia patients (35 male and 42 female patients, age 45.8±11.4 years) resulted in an improvement in symptoms four weeks later. Additionally, PET SUV revealed a statistically significant correlation with the scores for factors such as severity, disability, and pain before commencing treatment [31]. A positive correlation was observed between the number of hypermetabolic muscles and TWSTRS scores (total: r=0.531; severity: r=0.509; disability: r=0.445; pain: r=0.264). Similarly, a positive correlation was observed between the highest SUVmax of the hypermetabolic muscles and TWSTRS scores (total: r=0.39; severity: r=0.289; disability: r=0.327) [31].

*Methoxyisobutyl Isocyanide* *Scintigraphy*

Several studies have reported the utility of technetium-99m-methoxyisobutyl isocyanide ([99mTc]MIBI). In a study of 18 healthy participants (eight male and 10 female participants, age 38.7±13.1 years) and 18 patients with dystonia (8 male and 10 female patients, age 42.4±12.3 years), MIBI was used for the imaging of the healthy participants based on clinical findings. A comparison between the MIBI uptake and needle EMG findings yielded a Kappa value of 0.77. The sensitivity, specificity, and diagnostic coincidence rates of [99mTc]MIBI were 95.4%, 80.3%, and 90.8%, respectively [32]. Muscles that were positive for [99mTc]MIBI uptake were injected with botulinum toxin, and the TWSTRS score was evaluated two weeks, one month, three months, and six months later. No significant differences were observed between the healthy participants and the dystonia group after two weeks and one month; however, high scores were observed in both groups after three months and six months [32], indicating the duration of botulinum toxin efficacy. A comparison between 24 patients with cervical dystonia (7 male and 17 female patients, age 44.8±12.9 years) and 10 healthy participants in an evaluation of 227 muscles revealed that MIBI achieved a sensitivity, specificity, and area under the curve of 93.2%, 88.5%, 100%, and 0.908, respectively, for the sternocleidomastoid and elevator scapulae muscles [33]. Evaluation of 83 dystonic muscles of 60 patients with cervical dystonia (22 male and 38 female patients, age 47.3±13.4 years) revealed a total agreement rate of 94.0% between EMG and MIBI single-photon emission computed tomography (SPECT). The positive and negative agreement rates were 94.5% and 92.9%, respectively, with a kappa value of 0.866 [34]. The muscle/background ratio (MBR) and thickness of the obliquus capitis inferior muscle exhibited a positive correlation value of 0.330 [34]. In another study of 122 patients with cervical dystonia, the patients were divided into two groups: 53 patients underwent clinical evaluation and SPECT-CT (study group; 20 male and 33 female patients, age 45.4±14.4 years) and 55 patients underwent clinical evaluation only (control group; 20 male and 35 female patients, age 47.4±14.9 years). Muscles injected with botulinum toxin in the study group were identified using SPECT-CT with MIBI and clinical evaluation, whereas those injected with botulinum toxin in the control group were identified using clinical evaluation. Notably, symptom improvement was significantly better in the study group after one month. Furthermore, the outcomes of the SPECT-CT group were better after two weeks and three months [35]. A longer interval between the botulinum toxin injection and reinjection was observed in the study group (159.1±28.6 days vs. 141.8 ± 51.0 days) [35].

These findings indicate that FDG-PET and MIBI scintigraphy are useful for the diagnosis and treatment of dystonia. We show the data of FDG-PET and MIBI scintigraphy for the diagnosis of dystonia in Table 1.

**Table 1:** The data of FDG PET and MIBI scintigraphy for diagnosis of dystonia PET: positron emission tomography; SPECT: single-photon emission computed tomography; FGD: fluorodeoxyglucose; MIBI: methoxyisobutyl isonitrile

Number of patients	Sex	Modality	Sensitivity	Specificity	Reference number
6	Male patients: 6	PET-CT	100%	NA	[27]
10	Male patients: 5; Female patients: 5	PET-CT	76%	92%	[28]
18	Male patients:8; Female patients:10	SPECT-CT	95.4%	80.3%	[32]
24	Male patients: 7; Female patients: 17	SPECT-CT	93.2%	88.5%	[33]

Polymyositis/dermatomyositis

Polymyositis/dermatomyositis is characterized by autoimmune inflammation in the muscles. Physical examinations, blood tests, and MRI are used to diagnose polymyositis/dermatomyositis in routine practice. However, some studies have reported the utility of nuclear medicine for the diagnosis of polymyositis/dermatomyositis.

Fluorodeoxyglucose PET and Muscle Accumulation

A retrospective study involving 24 patients (eight male and 16 female patients, aged between 17 and 75 years, 11 patients with polymyositis/dermatitis, and 13 patients with dermatomyositis) showed a stronger accumulation of FDG in the muscle than in the liver on PET-CT examination in 33% of patients (8/24). The sensitivities of biopsy, EMG, and MRI were 100% (17/17), 72.6% (16/22), and 57% (12/21), respectively. These findings indicate that PET-CT has a low sensitivity [36]. Another retrospective study involving 20 patients (four male and 16 female patients, aged between 36 and 79 years, five patients with polymyositis/dermatitis/dermatitis, and 15 patients with dermatomyositis) reported that the SUVmax of distal muscle accumulation was 1.05 and 0.69 in the polymyositis/dermatitis and healthy (20 age- and sex-matched normal controls) groups, respectively, indicating a statistically significant difference. Statistically significant correlations were observed between the SUVmax and neural score (r = 0.49), creatine kinase (CK) (r = 0.54), and aldolase (r = 0.64) [37]. A retrospective study of 33 patients with pure polymyositis/dermatitis overlap disease, and mixed connective tissue disease (10 male and 23 female participants, mean age 56±17.9 years, involving) reported that the mean SUVmax in the proximal muscles exhibits a statistically significant correlation with the number of muscles with uptake (visual evaluation: r=0.87, total historical score: r=0.60) [38]. Another retrospective study of 24 patients with dermatomyositis (seven male and 17 female participants; median age 63 years) revealed that the ratio of SUVmax in the distal muscle to SUVmean in the liver (SUVprox/SUVliv) was higher than that in the longissimus muscle to SUVmean in the liver (SUVmlt/SUVliv). The ratio between SUVmax in the proximal muscles and SUVmax in the musculus longissimus thoracis (SXUVprox/SUVliv) was 1.73, with a sensitivity and specificity of 50% and 83.3%, respectively [39]. In 28 patients with polymyositis/dermatitis (four male and 24 female participants, age 44-77 years, nine patients with polymyositis, and 17 patients with dermatomyositis) and 28 healthy individuals. The SUVmax and SUVmean were 1.35±0.42 (normal 0.96±0.76) and 1.81±0.61 (normal 0.97±0.09), respectively, with a statistically significant difference (FDG PET/CT in patients with polymyositis/dermatitis: correlation with serum muscle enzymes) [40].

*Fluorodeoxyglucose* *PET and Lung Accumulation*

Several important studies have been conducted on lung diseases. Studies evaluating asymptomatic but rapidly progressive patients with anti-MDA5 antibody-positive polymyositis/dermatitis complicated by interstitial pneumonia with poor prognosis are underway. A retrospective study involving 26 MDA5-positive patients (eight male and 18 female patients, age 55.92±7.68 years) and 43 MDA5-negative patients (20 male and 23 female, age 56.88±13.65 years) with polymyositis/dermatitis revealed that the median SUVmax for pulmonary uptake was 0.72 and 0.58 in the MDA5-positive and MDA5-negative groups, respectively. The spleen uptake was 2.61 and 2.24 in the MDA5-positive and MDA5-negative groups, respectively. The bone uptake was 2.71 and 2.58 in the MDA5-positive and MDA5-negative groups, respectively, indicating statistically significant differences [41]. A recent study evaluating 62 patients with dermatitis (16 male and 46 female patients, age 55.4±12.7 years) revealed that in addition to cancer uptake, high FDG uptake by the pulmonary artery is a poor prognostic factor (hazard ratio (HR), 7.59; 95% confidence interval (CI), 2.08-27.76) [42]. This study reported that the presence or absence of interstitial pneumonia on high-resolution computed tomography is not a prognostic factor [42] and that the amount of FDG refracts the metabolic activity of the inflammatory cells; therefore, the prognosis with high FDG uptake in the pulmonary artery is poor [42]. Similarly, high pericardial fat is also a poor prognostic factor (HR, 5.86; 95% confidence interval (CI), 1.77-19.42) [37]. Moreover, in 22 patients (9 male and 13 female patients, age 38-69 years), a positive correlation between ^18^F-FDG uptake and SUVmax in the lung showed a visual score (0-12) for interstitial lung disease (r=0.565 and Krebs von den Lungen 6 (KL-6): r=0.476) [43]. Furthermore, FDG uptake and SUVmax in the muscle were positively correlated with CK (r=0.47817) [43]. These findings indicated that FDG uptake in the lungs is an important biomarker of interstitial lung disease.

BMIPP/Tl Scintigraphy and Polymyositis/Dermatitis/Dermatomyositis

Okada et al. conducted a retrospective study of 26 patients (three male and 23 female participants, age 53.3±15.3 years) with new-onset polymyositis/dermatitis to evaluate cardiac damage in these patients using 123I-BMIPP (BMIPP) and 201TlCl(Tl) scintigraphy. Notably, BMIPP/TlCL mismatches were observed in 12 (50%) patients. Concurrent echocardiography revealed that the end-diastolic diameter of the heart was 45 mm and 42.5 mm, the end-systolic diameter was 29.5 mm and 25 mm, and the EF was 63.5% and 71.5% in the mismatched and non-mismatched groups, respectively. This differentiation indicates microischemia in polymyositis/dermatitis [44]. Therefore, FDG PET and BMIPP/Tl scintigraphy can be used to diagnose and treat polymyositis and dermatitis.

Polymyalgia rheumatica

Polymyalgia rheumatica is an inflammatory disease characterized by fever, fatigue, and pain in the neck, shoulders, hips, and femurs. The diagnosis was made by physical examination, blood tests, and MRI, which has been used to diagnose polymyalgia rheumatica in routine clinical practice. However, some studies have reported on the utility of nuclear medicine. Some studies have reported the utility of FDG-PET for diagnosis.

Initial Research

Initial reports revealed FDG accumulation in four out of five patients with polymyalgia rheumatica [45]. Another study revealed FDG accumulation in 12 of 13 patients with polymyalgia rheumatica (two male and 11 female patients, aged between 48 and 77 years) [46]. These reports indicate the utility of FDG-PET for the diagnosis of polymyalgia rheumatica.

Disease-Specific Sites in FDG-PET

Fluorodeoxyglucose accumulation was observed in the shoulder (94%), hip (89%), and the spinous process of the vertebrae in 35 patients (16 male and 20 female patients, mean age 685±7.2 years) [47]. The FDG accumulation was observed at vascular sites in 11/35 patients (31%) [48]. High-frequency FDG accumulation was observed in the ischial tuberosities, greater trochanters, and lumbar spinous processes in 40 patients with polymyalgia rheumatica in another study [48]. The sensitivity and specificity for the diagnosis of polymyalgia rheumatica were 85.7% and 88.2% for diagnosis when ≥2 FDG accumulation was observed at the ischial tuberosities, greater trochanters, and lumbar spinous processes [48]. Accumulation at the articular/periarticular region was observed in 88.1% of patients (shoulders: 86.6%, sternoclavicular joints: 46.3%, and hips: 70.1%) in a study involving 67 patients (29 male and 28 female patients, median age 70 years) [49]. One, two, and three positive locations were observed in 16.4%, 28.4%, and 49.3% of cases, respectively [49]. The accumulation of extra-articular synovitis was observed in 76.1% of the patients (cervical interspinous bursae, 19.4%; lumbar interspinous bursae, 56.7%; ischiogluteal bursae, 52.2%; and prepubic bursae, 7.5%) [49]. Vascular involvement (large arteries) was observed in 40.1% of cases [49].

Sensitivity and Specificity of FDG-PET

A study involving 22 patients (13 male and nine female patients, mean age 68.3±6.3 years) revealed that the frequency of FDG uptake more than one is next: shoulder: 100%, hip: 86.4%, trochanteric: 100%, ischial: 95.4%, interspinous: 81.8%, knee: 76.2%, and wrist/hand: 66.7%) [50]. The sensitivity and specificity for the diagnosis of polymyalgia rheumatica were 85.7% and 88.2%, respectively, when >2 FDG accumulation was observed in the ischial tuberosities, greater trochanters, and lumbar spinous processes [48].

A study involving 33 patients with polymyalgia rheumatica (18 male and 15 female patients, age 68.6±7.4 years) and 132 control cases (72 male and 50 female patients, age 68.2±7.3 years) revealed that the sensitivity and specificity for detecting polymyalgia rheumatica based on FDG uptake of ≥1 at each musculoskeletal site are as follows: peri-articular shoulder, 97%/18.2%; glenohumeral joint, 72.7%/95.4%; acromioclavicular joint, 100%/33.6%; sternoclavicular joint, 87.9%/43.2%; interspinous bursa, 90.1%/82.6%; peri-articular hip, 7%/37.7%; hip joint, 60.6%/80.0%; trochanteric bursa, 100%/24.6%; ischial tuberosities, 10%/41.6%; knee joint, 53.1%/46%; and posteromedial knee, 75%/80.1% [51]. The sensitivity and specificity for polymyalgia rheumatica based on FDG uptake of ≥1 at each musculoskeletal site in a study involving 39 patients with polymyalgia rheumatica (26 male and 13 female patients, median age 71 years) and 19 control individuals (13 male and six female patients, median age 59 years) are as follows: ischial tuberosities, 97.4%/52.6%; hips, 100%/42.1%; lumbar interspinous bursa, 92.3%/57.9%; sternoclavicular joints, 71.8%/84.2%; symphysis pubisentheses, 69.2%/98.95%; greater trochanters, 100%/10.5%; lumbar facet joints, 69.2%/89.5%; cervical interspinous bursa, 74.4%/73.8%; knee, 89.3%/33.3%; shoulders, 97.4%/10.5%; and Iliopectineal bursae, 43.6%/100% [52]. Thus, FDG accumulation was observed at disease-specific sites in patients with polymyalgia rheumatica, including the shoulder (peri-articular shoulder), hip, and ischial tuberosities. The number of accumulation sites was ≥2 or 3.

Disease Symptoms

Fluorodeoxyglucose uptake was observed in the spinous processes, hip, ischial tuberosity, and vascular sites in symptomatic and non-symptomatic groups in a study involving 29 steroid-naïve patients with polymyalgia rheumatica (18 male and 11 female patients; median age 76 years) [53]. The presence of symptoms did not affect FDG uptake.

*Timing​​​​​​​ of* ^18^ *F-FDG*

A study involving 67 patients with polymyalgia rheumatica (27 male and 40 female patients; mean age 72 years) and 27 patients with other inflammatory diseases (13 male and 14 female patients; mean age 69 years) revealed that the initial PET/CT sensitivity and specificity were 86% and 63% (Leuven score) and 82% and 70% (dichotomous score), respectively. The sensitivity and specificity of PET/CT after long-term (eight weeks) prednisolone therapy were 39% and 54%, respectively. In contrast, PET/CT sensitivity and specificity following short-term prednisolone therapy were 60% and 66% (Leuven score) and 39% and 56% (dichotomous score), respectively [54]. A study involving 99 patients with a possible clinical diagnosis of polymyalgia rheumatica (67 diagnosed with polymyalgia rheumatica; 29 male and 38 female patients, median age 71 years, and 32 without polymyalgia rheumatica, 12 male and 20 female patients, median age 64 years) that used clinical and musculoskeletal scores based on PET before commencing glucocorticoid therapy reported improved diagnostic quality [55]. A study involving 101 patients with polymyalgia rheumatica (53 male and 48 female patients, age 68.6±10.9 years) and 97 controls (41 male and 56 female patients, age 67.7±9.4 years) revealed ^18^F-FDG uptake in the muscle (34 vs. 10%) [56]. Muscle inflammation was observed in almost 30% of patients with polymyalgia rheumatica, indicating that PET/CT should be performed before commencing treatment.

Differentiation Between Polymyalgia Rheumatica and Other Diseases

A study involving 37 patients with polymyalgia rheumatica (16 male and 21 female patients; median age 73 years) and 15 patients with immune checkpoint inhibitor-mediated polymyalgia rheumatica (nine male and six female patients; median age 71 years) revealed that the FDG uptake score was lower in the immune checkpoint inhibitor-mediated polymyalgia rheumatica group [57]. Another study involving 15 patients with polymyalgia rheumatica (five male and 10 female patients, aged 72 years) and seven patients with elderly onset rheumatoid arthritis (two male and five female patients, aged 77 years) revealed that patients with polymyalgia rheumatica exhibited high PET scores [58]. Another study involving 35 patients with polymyalgia rheumatica (14 male and 21 female patients; median age 71 years) and 27 patients with spondylarthritis (15 male and 12 female patients; median age 54 years) revealed that FDG uptake in the enthesis/bursae was more prevalent in patients with polymyalgia rheumatica than in those with spondylarthritis (ischial tuberosities, 88.6% vs. 48.1%; interspinous processes, 91.4% vs. 51.9%) [59].

Standard Uptake

The diagnosis of polymyalgia rheumatica is primarily based on a visual evaluation. However, some studies have reported SUV. A PET study involving 78 patients with polymyalgia rheumatica (20 male and 50 female patients, age 67.54±8.09 years) and 123 individuals without polymyalgia rheumatica (60 male and 61 female patients, age 65.23±8.90 years) evaluated the SUV index (the SUVmax value of the region of interest (ROI)/liver mean SUV) at the regions of interest comprising the ischial tuberosity, interspinous bursa, periarticular hip, and symphysis pubis enthesis. The area under the curve of the characteristic site SUV index was 0.930, and the best cutoff value was 1.685, with a sensitivity of 84.6% and a specificity of 92.6% [60]. In 85 patients with new onset polymyalgia rheumatica (34 male and 51 female patients, age 70.7±8.6 years) and 75 individuals without polymyalgia rheumatica (37 male and 38 female, age 65.2±10.9 years) revealed uptake in the whole body (11.3±3.3 vs 0.9±1.1) and high SUVmax (5.4±1.3 vs. 2.7±0.4) in the patients [61]. The sensitivity of FDG-PET was 86%, specificity was 85.5%, and SUVmax at 17 points was 2.168 in 57 patients with polymyalgia rheumatica. The sensitivity and specificity were 77.2% and 77.5%, respectively, for the differential diagnosis (polymyalgia rheumatica and other inflammatory rheumatism) [62].

Therapy

Among 103 patients with polymyalgia rheumatica (30 male and 73 female patients, age 72.4±9.0 years) in a previous study, almost 50% of patients received FDG-PET-guided therapy owing to resistance to steroid therapy, and the true polymyalgia rheumatica rate was almost 70% (large vessel vasculitis: 15.5% and neoplasms: 4.8%) [63]. However, a study involving 14 steroid-sensitive patients with polymyalgia rheumatica (seven male and seven female patients, age 65.5±11.4 years) and 61 steroid-resistant patients with polymyalgia rheumatica (20 male and 41 female patients, age 69.9±10.5 years) revealed a similar FDG uptake pattern [63]. The FDG uptake pattern is not necessarily useful for determining steroid response [64]. Magnetic resonance imaging studies evaluating patients with high intensity in the greater trochanter, acetabulum, ischial tuberosity, and/or symphysis pubis have revealed high CRP and high interleukin-6 (IL-6) levels. However, a good glucocorticoid response and improvement in fatigue and function were observed after treatment [65].

Diagnostic Flow

A previous study revealed the usefulness of PET/CT in the diagnosis of polymyalgia rheumatica [66]. In this flow, the following specific FDG uptake pattern indicates the probability of polymyalgia rheumatica: FDG uptake at ≥ 2 sites in the positive interspinous bursa and FDG uptake at ≥2 sites in the trochanteric bursa, if FDG uptake at ≥2 sites in the positive interspinous bursa, is false [66]. Thus, FDG-PET is a useful modality for the diagnosis and treatment of polymyalgia rheumatica.

These findings indicate that FDG-PET is useful for diagnosing polymyalgia rheumatica.

## Conclusions

Ultrasonography, MRI, and FDG are valuable tools for assessing muscle health and diagnosing muscle-related diseases. Although ultrasonography and MRI provide detailed anatomical and functional information, FDG-PET offers insights into muscle metabolism and disease-specific uptake patterns. An FDG-PET scan is useful for the diagnosis of dystonia, polymyositis/dermatomyositis, and polymyalgia rheumatica; however, in this study, the sample size was small, so we did not use a meta-analysis or systematic review, and we used a narrative review.

There were limited reports regarding radiation exposure, contrast resolution, and spatial resolution, we thus expect further increased reporting, and that a meta-analysis and systematic review will be performed. Moreover, we expect a clinical trial to compare MRI, especially DWIBS, and PET in terms of medical costs and diagnostic utility.
